# Pyrexia of Unknown Origin in a Young Male: Unmasking Melioidosis in a Tuberculosis-Endemic Setting

**DOI:** 10.7759/cureus.64024

**Published:** 2024-07-07

**Authors:** Mokkarala Satya Vamsi Krishna, Suja Lakshmanan, Vaasanthi Rajendran, N. Senthil, Irfan Ismail Ayub

**Affiliations:** 1 General Medicine, Sri Ramachandra Institute of Higher Education and Research, Chennai, IND; 2 Pulmonary and Critical Care Medicine, Sri Ramachandra Institute of Higher Education and Research, Chennai, IND

**Keywords:** ebus-tbna, nested-pcr, tuberculosis, pyrexia of unknown origin (puo), systemic melioidosis

## Abstract

A young male, plantation worker from Southeast Asia, presented with a non-productive cough, intermittent high-grade fever with chills, and significant weight loss over two months. Prior investigations were non-contributory, despite various antibiotics, his symptoms persisted. Physical examination and routine investigations, including an extensive microbiological workup for fever were non-contributory. A positron emission tomography-computed tomography (PET-CT) scan performed for pyrexia of unknown origin (PUO) revealed pulmonary consolidation, mediastinal lymphadenopathy, and splenic microabscesses. Material aspirated via endobronchial ultrasound-guided transbronchial needle aspiration (EBUS-TBNA) from the left interlobar lymph node was positive for *Burkholderia pseudomallei* on conventional nested polymerase chain reaction (PCR), confirming a diagnosis of melioidosis. Following appropriate antibiotic therapy, there was a complete resolution of symptoms. This case underscores the diagnostic challenges and the need for advanced techniques in identifying melioidosis, which can mimic tuberculosis.

## Introduction

Melioidosis is an emerging disease in South Asia caused by the Gram-negative bacillus *Burkholderia pseudomallei*. It is found in soil and water. Humans can be infected through inhalation, ingestion, or contact with contaminated soil or water, especially in agricultural and construction settings and humid climates. South Asia contributes to 44% of the global burden [1], but exact incidence rates are unclear due to lack of awareness, underreporting, and misdiagnosis [2]. Major risk factors include diabetes mellitus, chronic renal, liver, and lung diseases, alcohol abuse, occupational exposure, and chronic skin ulcers [3]. The incubation period is about one to 21 days [4].

The disease ranges from asymptomatic to chronic infection, manifesting as localized skin infections to severe septicemia and multi-organ abscesses. Pneumonia, including pleural effusion, is the most common clinical manifestation, followed by bacteremia and septic shock, genitourinary infections, prostatic abscess, skin or soft tissue infections, bone or joint infections, neurological melioidosis, splenic abscesses, other intraabdominal infections, liver abscesses, and pericardial effusion [5]. Treatment requires prolonged antibiotics due to intrinsic resistance patterns, and case fatality rates can be as high as 10-50% without appropriate therapy [5].

Southeast Asia has 45% of the world's annual tuberculosis incidence [6]. Melioidosis and tuberculosis share similar transmission routes, risk factors, and clinical pictures, but their investigations and treatments differ. Hence, suspecting "emerging melioidosis" is crucial despite the endemicity of tuberculosis.

## Case presentation

A young male in his early 20s, working as a plantation worker in Southeast Asia, presented with a dry cough for two weeks, intermittent high-grade fever with chills, and weight loss of 5 kg over the last two months. Despite receiving various antibiotics at local hospitals, his symptoms persisted. He reported close contact with his mother and brother, who were diagnosed and treated for tuberculosis in the past two years.

His physical examination was unremarkable. Blood investigations revealed anemia, raised erythrocyte sedimentation rate, and C-reactive protein levels. Total leukocyte and platelet counts were normal. He had normal liver, renal, and thyroid biochemistry (Table 1). The urine examination was unremarkable. A comprehensive workup for fever, including serologies for dengue, scrub typhus, and leptospirosis, and quantitative buffy coat (QBC) for malaria, blood and urine cultures were all negative. Viral screening for human immunodeficiency virus (HIV), and hepatitis B and C were negative. The tuberculin skin test was negative. Anti-nuclear antibody (ANA) by immunofluorescence and rheumatoid factor were also negative.

**Table 1:** Basic blood workup of the patient.

Laboratory parameters	Patient’s value	Reference range
Hemoglobin	10 g/dL	12-17 g/dL
Total leukocyte count	6,240/mm^3^	4,000-11,000/mm^3^
Platelet count	334,000/mm^3^	150,000-450,000/mm^3^
Erythrocyte sedimentation rate	47 mm/h	0-15 mm/h
C-reactive protein	4.3 mg/dL	<1 mg/dL
Serum creatinine	0.8 mg/dL	0.8-1.3 mg/dL
Blood urea nitrogen	8 mg/dL	7.9-20.1 mg/dL
Free thyroxine (free T4)	1.17 ng/dL	0.93-1.7 ng/dL
Free triiodothyronine (free T3)	2.14 pg/mL	2-4.4 pg/mL
Thyroid-stimulating hormone (TSH)	2.140 μIU/mL	0.27-4.2 μIU/mL
Total bilirubin	1 mg/dL	0.3-1.2 mg/dL
Serum alanine aminotransferase (ALT)	42 IU/L	<50 IU/L
Serum aspartate aminotransferase (AST)	29 IU/L	<50 IU/L
Total protein	7 g/dL	6.6-8.3 g/dL
Serum albumin	4 g/dL	3.5-5.2 g/dL

The chest radiograph was normal. Abdominal ultrasonography revealed no significant abnormalities. An electrocardiogram (ECG) showed normal sinus rhythm. A transthoracic echocardiogram showed normal structural chambers and valves, no regional wall motion abnormality, with an ejection fraction of 63%, and no evidence of vegetation.

**Figure 1:**
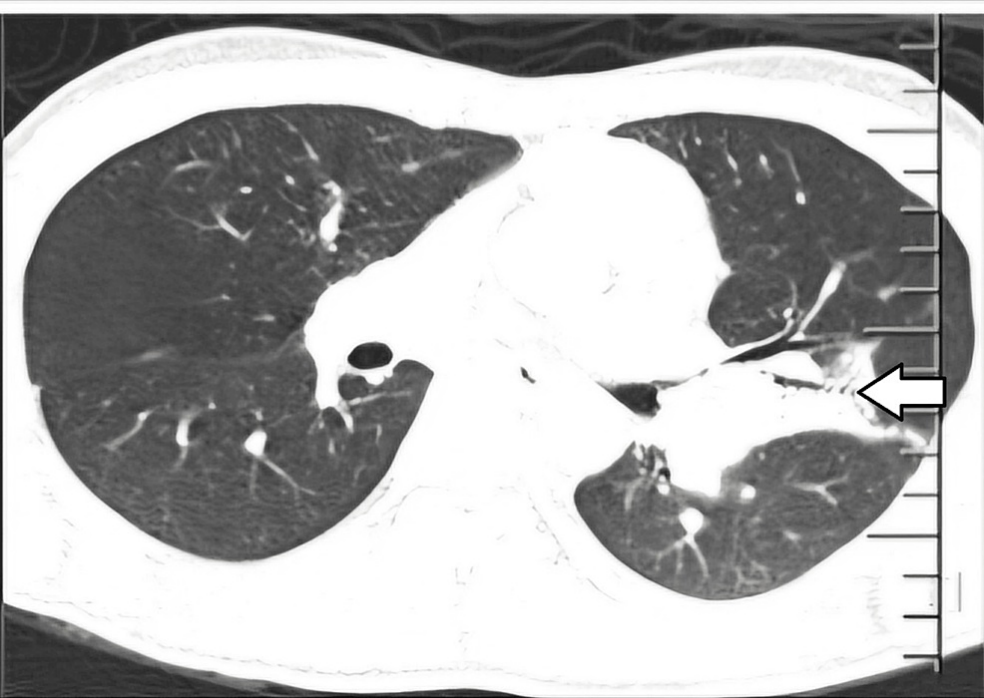
PET-CT showing consolidation of upper lobe of left lung (arrow).

**Figure 2:**
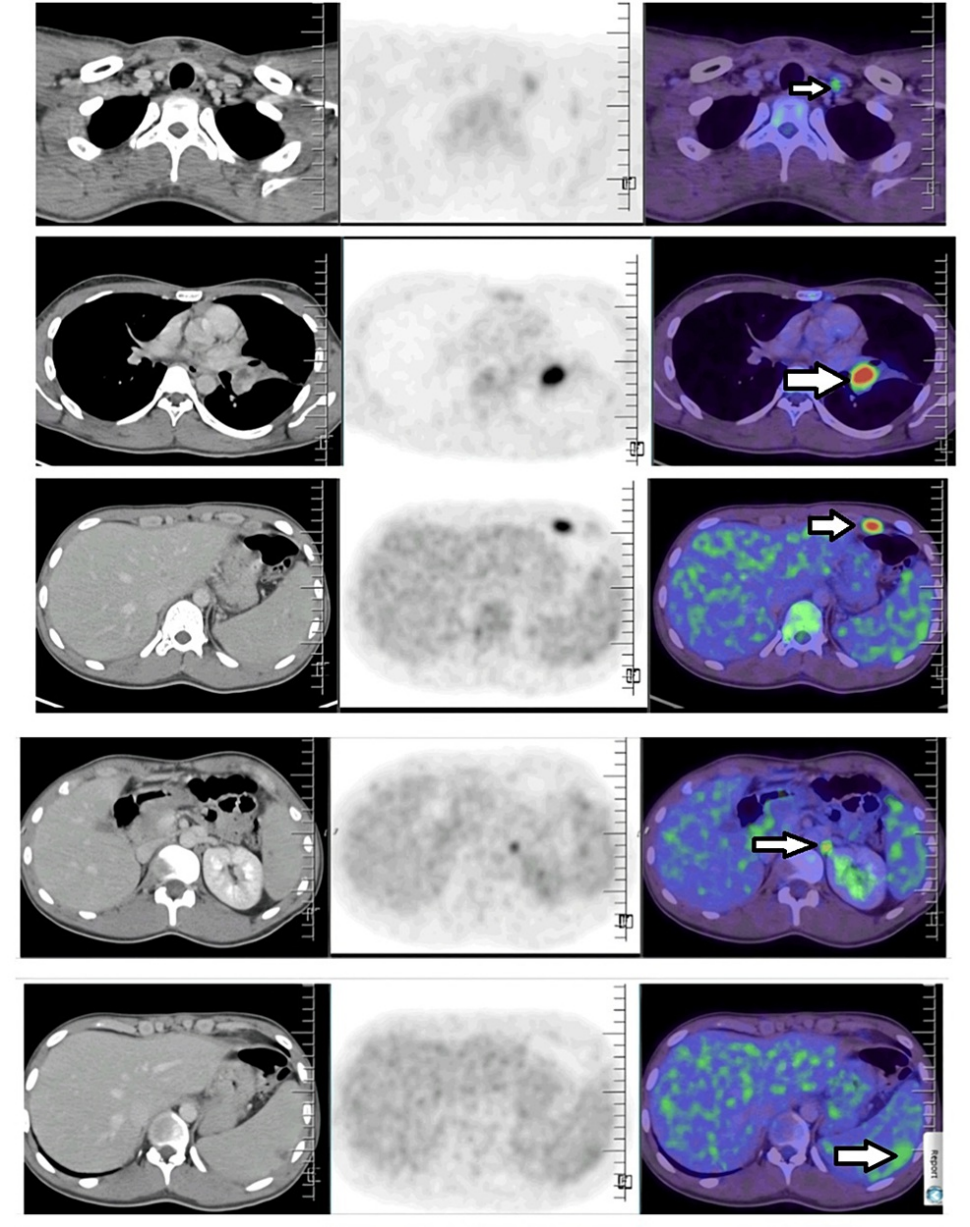
PET-CT scan showing FDG avid lymph nodes and splenic microabscess (arrows). FDG: fluorodeoxyglucose

Due to inconclusive microbiological investigations and persistent fever spikes, a positron emission tomography-computed tomography scan with 18-fluorodeoxyglucose (18-FDG PET-CT) was performed. It revealed pulmonary consolidation in the left upper lobe (Figure 1), lymphadenopathy involving bilateral supraclavicular, prevascular, left internal mammary, aortopulmonary window, left hilar, left epiphrenic, and para-aortic abdominal regions, and mild splenomegaly (12.1 cm) with multiple focal hypodense lesions (Figure 2).

**Figure 3:**
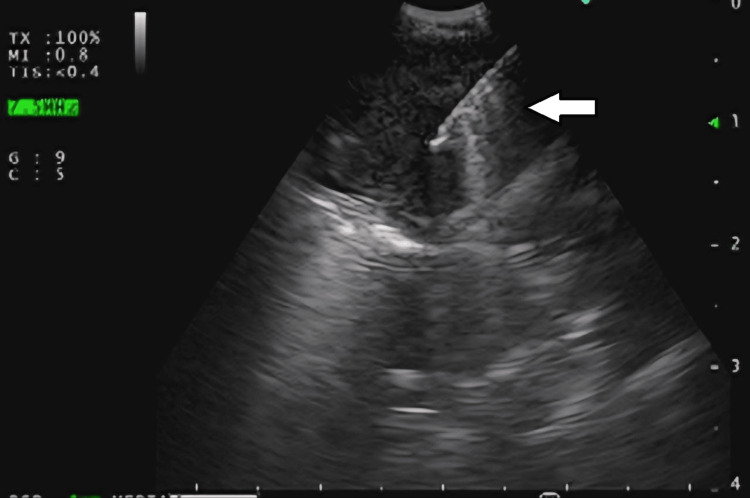
EBUS-TBNA of left interlobar lymph node. EBUS-TBNA: endobronchial ultrasound-guided transbronchial needle aspiration

**Figure 4:**
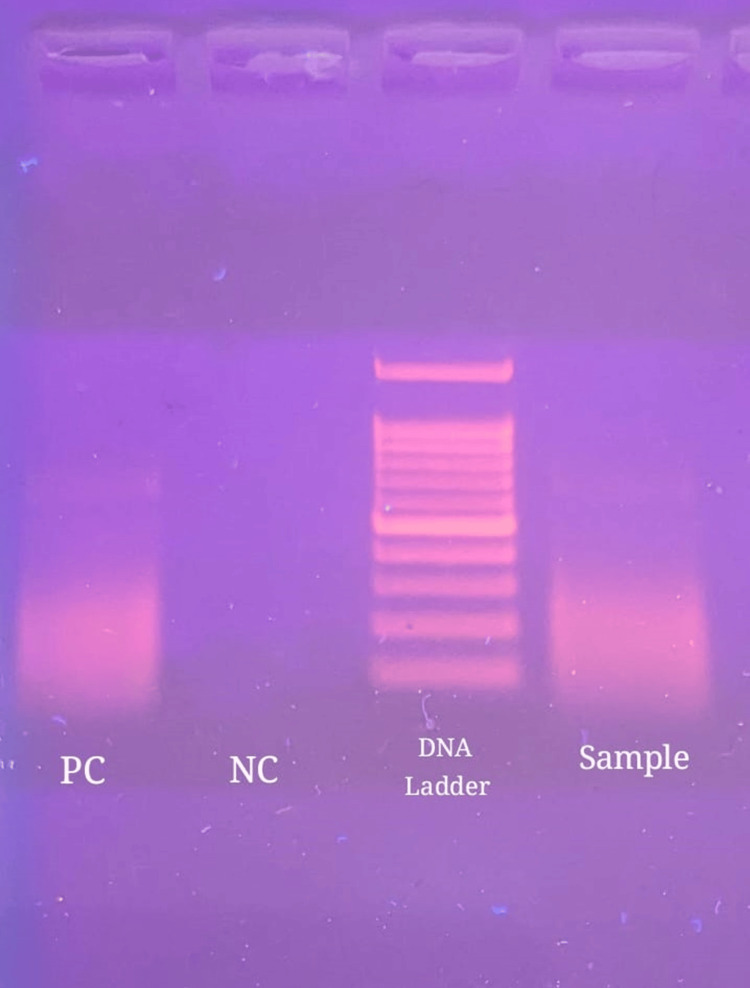
Gel image from PCR analysis showing the presence of Burkholderria pseudomallei DNA in the sample, similar to that of a positive control (PC). PC: positive control; NC: negative control

As the supraclavicular and abdominal lymph nodes were not amenable to biopsy, he was subjected to endobronchial ultrasound-guided transbronchial needle aspiration (EBUS-TBNA) of the mediastinal lymph nodes, along with bronchoscopy and bronchoalveolar lavage (BAL) from the left upper lobe (Figure 3). Material aspirated via EBUS-TBNA from the left interlobar node was positive for *Burkholderia pseudomallei* on conventional nested polymerase chain reaction (PCR), targeting the 16s-23s spacer region of *Burkholderia pseudomallei* (Figure 4).

Stains for tuberculosis (acid-fast stain) and fungus, GeneXpert MTB (Sunnyvale, CA: Cepheid), and cultures for tuberculosis, fungi, and bacteria all returned negative. Needle core biopsy material aspirated from the left interlobar node via EBUS-TBNA revealed necrotizing granulomatous inflammation on histopathological examination of the tissue. BAL fluid was negative for microbiological investigations for tuberculosis, fungi, and bacteria as well.

He was diagnosed with non-critical melioidosis with pneumonia and significant lymphadenopathy, and without neurological involvement, he was initiated on intravenous ceftazidime 2 g every 8 h during the intensive phase. He became afebrile after five days of antibiotic therapy initiation. After completing four weeks of intensive phase, he was switched over to the eradication phase of treatment, with oral co-trimoxazole (with 320 mg of trimethoprim component) twice a day for three months. On follow-up, he had completed the three-month course of co-trimoxazole and had resumed normal activities two months earlier.

## Discussion

Pyrexia of unknown origin (PUO) is a condition where a patient has a fever higher than 38.3°C (101°F) for at least three weeks and the cause is inconclusive despite thorough investigations. It may resolve spontaneously or with treatment, and can often pose a formidable diagnostic challenge [7]. The causes of PUO include infection, inflammation, malignancy, or miscellaneous conditions. Infections are still the leading cause of PUO, with tuberculosis being the single largest cause (28%), particularly in Southeast Asian countries [8]. Once tuberculosis is excluded during investigations performed for workup of PUO, physicians often find themselves in a tight spot trying to identify the other infective causes of PUO.

Melioidosis can present as asymptomatic to chronic infection, and from localized skin infections to severe septicemia and multi-organ abscesses. Pneumonia, including pleural effusion, is the most common manifestation and is seen in nearly half of the cases [5]. It can present acutely (within two months) or sub-acute to chronically (more than two months), mimicking community-acquired pneumonia and tuberculosis, respectively. Upper lobe consolidation, common in chronic forms, can also be seen in acute forms. Mediastinal lymphadenopathy is also common. Chest imaging can show small or no infiltrates, diffuse or patchy, lobar or multi-lobar consolidation, necrotizing lesions, pleural effusions, and cavitating lesions (like tuberculosis).

Confirmation of the diagnosis is typically done by culturing blood, pus, or other body fluids, which is considered the gold standard but is positive in only 60% of cases [4]. The relatively poor sensitivity of culture is attributed to low bacterial load or prior antibiotic use. Also, Burkholderia colonies can be mistaken for other organisms, potentially leading to mistaken diagnoses [9].

Although culture is diagnostic, it is time-consuming. Therefore, accurate but quicker methods like PCR are needed for early detection and treatment. Conventional nested PCR targeting the 16S-23S spacer region was used here. The procedure involves adding primers to the extracted plasmid DNA of *Burkholderia pseudomallei* followed by two cycles of PCR amplification. The resulting amplification product is then analyzed using 2% agarose gel electrophoresis and a gel documentation unit. A band size of 251 base pairs (bp) confirms the presence of the spacer region, indicating a positive result [10]. The 16S primers are specific to *Burkholderia pseudomallei*, minimizing misidentification [11]. This method is valuable for differentiating melioidosis from other microorganisms, including tuberculosis. There have been cases reported in the literature where PCR-based testing has helped to identify Burkholderia infection in patients with a clinical suspicion of tuberculosis [12,13].

Treatment for melioidosis requires prolonged antibiotics in two phases. Intravenous antibiotics like ceftazidime, meropenem, or imipenem are administered during the intensive phase for two to eight weeks. Ceftazidime is usually dosed at 50 mg/kg up to 2 g every 6-8 h [14]. After completion of the intensive phase, oral antibiotics such as co-trimoxazole, doxycycline, or amoxicillin-clavulanate are administered for 3-6 months during the eradication phase, with co-trimoxazole preferred at 320 mg of trimethoprim twice daily for adults over 60 kg [14]. Challenges include prolonged treatment, adherence, follow-up, and risks of relapse and reinfection [15]. Prevention involves avoiding direct soil and water contact and minimizing outdoor activities, especially during the rainy season. Currently, no vaccines are available [16].

## Conclusions

This study underscores the diagnostic challenges posed by melioidosis, which can mimic tuberculosis. Advanced diagnostic techniques, such as EBUS-TBNA and PCR, were crucial in confirming the diagnosis after initial inconclusive investigations. Prompt initiation of appropriate antimicrobial therapy with ceftazidime and co-trimoxazole led to rapid clinical improvement and successful treatment outcomes. Clinicians in endemic regions should maintain a high index of suspicion for melioidosis.
